# CRACking the Molecular Regulatory Mechanism of SOCE during Platelet Activation in Thrombo-Occlusive Diseases

**DOI:** 10.3390/cells11040619

**Published:** 2022-02-10

**Authors:** Patrick Münzer, Oliver Borst

**Affiliations:** 1 Department of Cardiology, Angiology and Cardiovascular Medicine, University of Tübingen, 72076 Tübingen, Germany; 2 DFG Heisenberg Group Thrombocardiology, University of Tübingen, 72076 Tübingen, Germany

**Keywords:** platelets, STIM1, Orai1, SOCE, CRAC, thrombosis, myocardial infarction, ischemic stroke, pulmonary embolism

## Abstract

Thrombo-occlusive diseases such as myocardial infarction, ischemic stroke and deep vein thrombosis with subsequent pulmonary embolism still represent a major health burden worldwide. Besides the cells of the vasculature or other hematopoietic cells, platelets are primarily responsible for the development and progression of an occluding thrombus. The activation and function of platelets crucially depend on free cytosolic calcium (Ca^2+^) as second messenger, which modulates platelet secretion, aggregation and thrombus formation. Ca^2+^ is elevated upon platelet activation by release of Ca^2+^ from intracellular stores thus triggering of the subsequent store-operated Ca^2+^ entry (SOCE), which is facilitated by Ca^2+^ release-activated channels (CRACs). In general, CRACs are assembled by the pore-forming unit Orai in the plasma membrane and the Ca^2+^-sensing stromal interaction molecule (STIM) in the endoplasmic reticulum after the depletion of internal Ca^2+^ stores. In the last few years, there is a growing body of the literature demonstrating the importance of STIM and Orai-mediated mechanism in thrombo-occlusive disorders. Thus, this review provides an overview of the recent understanding of STIM and Orai signaling in platelet function and its implication in the development and progression of ischemic thrombo-occlusive disorders. Moreover, potential pharmacological implications of STIM and Orai signaling in platelets are anticipated and discussed in the end.

## 1. Introduction

Thrombo-occlusive disorders of the arterial and venous vascular system such as myocardial infarction, ischemic stroke and venous thromboembolism with accompanying complications still represent a major cause of morbidity and mortality in the western civilization [1]. Thereby, platelet functions such as adhesion, secretion and aggregation are pivotal for primary hemostasis at sites of vascular injury, but are also highly involved in pathological thrombus formation, inflammatory processes and atherogenesis [2]. In particular, the exposure of subendothelial collagen after atherosclerotic plaque rupture or endothelial injury in the arterial system, as well as low shear flow in the venous system, induces initial platelet adhesion and activation [2,3]. Upon initial activation by subendothelial collagen, platelets release intracellular adenosine diphosphate (ADP), adenosine triphosphate (ATP) and serotonin, synthesize bioactive lipids such as thromboxane A_2_ (TxA_2_) or 12(S)-HETE and become pro-coagulant by exposure of phosphatidylserines, thus supporting the production of the most powerful platelet agonist, thrombin, by the coagulation cascade [4,5,6]. Altogether, all of these agonists act as autocrine or paracrine mediators to maintain platelet activation, recruit additional cells to the site of injury and finally initiate thrombus formation. However, despite the plurality of platelet agonists, platelet activation always results in a rise of intracellular Ca^2+^, which then acts as an important second messenger triggering further intracellular signaling pathways, inside-out signaling and finally culminates in thrombus formation [7]. Consequently, to avoid the formation of vessel-occluding pathological thrombi or accompanying ischemic tissue damages, but still maintain primary hemostasis, platelets require strict regulation mechanisms of their intracellular Ca^2+^ concentrations.

Of note, despite significant improvements in the prophylaxis and treatment of thrombo-occlusive disorders, current anti-platelet treatment regimens often impair primary hemostasis thus causing severe bleeding complications. These bleeding effects reflect our incomplete understanding of the complex Ca^2+^-dependent cellular and molecular mechanisms underlying thrombogenesis. However, although the details of Ca^2+^ homeostasis and signaling during platelet activation are complex, pharmacological intervention into the Ca^2+^ signaling of platelets could be a promising future target for the treatment of thrombo-occlusive disorders.

Thus, in this review, we present a brief summary of platelet physiology and intracellular signaling before providing an overview of the recent understanding of the molecular regulation mechanisms of SOCE-mediated processes, since this pathway represents the most essential mechanism for Ca^2+^ influx in platelets [8,9,10]. Afterwards, its implication in (pathological) thrombus formation will be attended and finally potential pharmacological approaches of SOCE inhibition in the treatment of thrombo-occlusive disorders will be discussed.

## 2. Platelet Physiology and Mechanisms/Pathways of Ca^2+^ Signaling

Platelets are critically involved in pathological thrombus formation. They are generated by cytoplasmic fragmentation of polyploid megakaryocytes mainly within sinusoids in the bone marrow [11,12], but also to a certain extent in the lung [13]. Arising from pluripotent stem cells, megakaryocytes undergo multiple rounds of endomitosis to amplify the genetic material under the stimulating control of the glycoprotein thrombopoietin (TPO). During this thrombopoiesis process platelet-specific proteins are accumulated, determining the later protein composition of the platelets. Consequently, megakaryocytes form pseudopodal extensions designated pro-platelets, which reach into the sinusoids of the bone marrow, leading to platelet fragmentation from the end of the megakaryocyte extensions by means of the capillary shear rates and the final platelet release in the blood flow [11].

Due to the described nature of the megakaryopoiesis and thrombopoiesis process, platelets are small anucleated cells with a diameter of 2–3 µm. They circulate the blood flow for approximately 10 days before being cleared from circulation by phagocytosis-like processes mostly in the liver and to a shorter extent in the spleen [14]. Although native platelets are activated by a wide variety of agonists, including (subendothelial) collagen, collagen-related peptide (CRP), thrombin, ADP or bioactive lipids [5,6,15], mostly two different intracellular signaling pathways exist in platelets. On the one hand, protease-activated receptors (PARs) such as PAR1 and PAR4 in humans or PAR3 and PAR4 in mice [16], as well as purinergic P2Y receptors are expressed on the platelet surface. Both receptor types belong to the G protein-coupled receptor (GPCR) family and mediate thrombin- or ADP-related platelet activation, respectively. The platelet immune receptor glycoprotein VI (GPVI), on the other hand, has an immunoreceptor tyrosine-based activation motif (ITAM) and mediates primarily collagen and/or CRP-dependent platelet activation [17]. GPVI-mediated ITAM signaling is restricted by a phosphoinositide 3-kinase (PI3K)-derived phospholipid interactome. Using a mass spectrometry approaches Durrant et al. just recently defined the dual adaptor for phosphotyrosine and 3-phosphoinositides (DAPP1/Bam32/PHISH) as a downstream target of GPVI signaling, which negatively regulates collagen-induced platelet activation since platelets from DAPP1 deficient mice showed increased granule secretion, α_IIb_β_3_ integrin activation and finally an enhanced in vitro thrombus formation [18,19].

Activation of both, GPCR and GPVI signaling pathways, result in a phospholipase C (PLC)-dependent hydrolysis of phosphoinositides (PI) [20] which is followed by the generation of inositol-1,4,5-triphosphate (IP_3_) and 1,2-diacyl-glycerol (DAG) [7]. While GPCRs utilize the PLC-ß isoform, the GPVI-dependent signaling pathway acts via the PLC-γ isoform. In both ways, the generated IP_3_ and DAG always cause extracellular Ca^2+^ influx across the plasma membrane, which is finally responsible for platelet granule release, integrin α_IIb_β_3_ activation, phosphatidylserine (PS) exposure, platelet aggregation, and thrombus formation [7].

**Figure 1:**
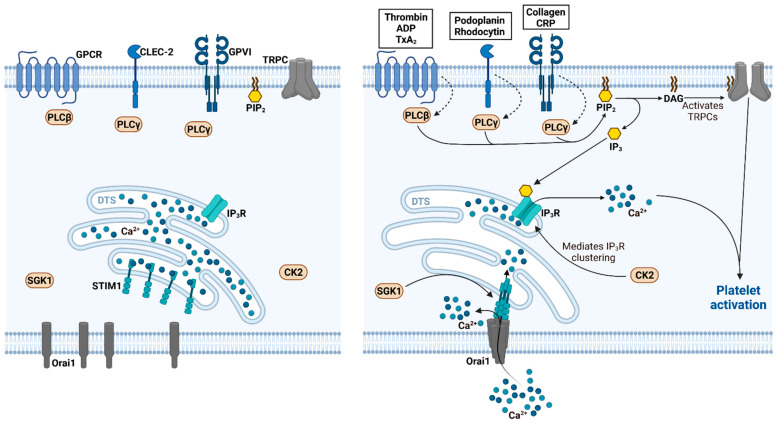
SOCE-mediated Ca^2+^ signaling in platelet activation. Left panel: In resting platelets, Ca^2+^ is bound to the intraluminal EF-hand domain of STIM1 thus impeding STIM1 clustering and subsequently CRAC formation. Right panel: Upon receptor-mediated platelet activation by a wide variety of agonists, the IP_3_R clustering/sensitization is supported by casein kinase 2 (CK2)-dependent processes and the PLC-dependent generation of IP_3_ causes a depletion of internal Ca^2+^ stores and the unloading of the EF hand of STIM1. Consequently, STIM1 clusters and initialize the assembly of the CRAC by clustering of the Orai1 subunits which is supported by the serum and glucocorticoid-regulated kinase 1 (SGK1). The assembly of the CRAC finally results in the influx of extracellular Ca^2+^ by means of the SOCE and platelet activation.

Thereby, DAG can directly induce Ca^2+^ influx by activating transient receptor potential cation channels (TRPCs) in the plasma membrane. While the TRPCs represent one of the largest ion channel families, platelets mainly express the isoforms TRPC1 and TRPC6, whereas especially TRPC6 is present in the plasma membrane and was thought to be the major channel for activation-dependent and non-selective extracellular cation influx in platelets [21]. However, TRPC1-deficient mice showed unaffected platelet functions [22], pointing to the involvement of another Ca^2+^-dependent mechanism of platelet activation. Indeed, severe immunodeficiency due to dysfunctional components of the SOCE in human patients is also frequently linked to abnormal platelet function [23]. In platelets, Ca^2+^ is mainly stored in the dense tubular system (DTS), which is the equivalent to the sarco- or endoplasmic reticulum (ER) in other cells, lysosome-like acidic Ca^2+^ stores and mitochondria [24]. Like in other cells, the store content and intracellular Ca^2+^ concentration in platelets are maintained by Ca^2+^-ATPases designated sarco-/endoplasmic reticulum Ca^2+^-ATPases (SERCAs), whereas different isoforms are located in the particular Ca^2+^ stores such as SERCA2b in the DTS or SERCA3 in lysosome-like acidic Ca^2+^ stores [25,26]. In general, IP_3_ especially mediates the release of Ca^2+^ from the internal DTS, since IP_3_ receptors (IP_3_Rs) are highly expressed on the surface of the DTS and facilitate a rapid Ca^2+^ release in platelets upon activation-dependent binding of IP_3_ [27]. In addition to the binding of IP_3_, the intracellular Ca^2+^ release is highly dependent on the clustering and activation of the IP_3_R, a mechanism which requires microtubule-associated linkage of the end binding protein EB3 in endothelial cells [28]. In this regard, the ubiquitous serine/threonine-selective protein kinase casein kinase 2 (CK2) was described to regulate tubulin polymerization and EB3 binding in platelets thus affecting IP_3_-induced intracellular Ca^2+^ release and thrombus formation [29]. Independent of the underlying regulatory mechanisms, the clearance of internal Ca^2+^ stores by IP_3_ however subsequently triggers the extracellular Ca^2+^ influx across the plasma membrane employing the molecular machinery of the SOCE [9] (Figure 1).

## 3. Components of the SOCE Machinery in Platelets

In non-excitable cells, the entry of Ca^2+^ is primarily mediated by the activation-dependent SOCE, which has been known since the early 1990s to be the most important mechanism for Ca^2+^ influx after store depletion in platelets [30,31]. However, only in 2005 and 2006 function-based genetic screens using RNA systematic interference revealed the stromal interaction molecule 1 (STIM1) and Orai1 as the crucial molecular components of the SOCE in lymphocytes [32,33,34]. Based on these findings, STIM1 and Orai1 were also identified as the major components of activation-induced Ca^2+^ signaling in platelets shortly afterwards [9,10,35].

The stromal interaction molecule 1 (STIM1) belongs to the highly conserved family of STIM proteins consisting of the two isoforms STIM1 and 2. Both isoforms are expressed in platelets [36], whereas STIM1 seems to be the more important isoform for platelet function [10]. In general, STIM1 undergoes conformational changes upon store depletion, multimerizes and form punctae in the DTS membrane, which subsequently interacts with its counterpart Orai1 in the plasma membrane [37]. STIM1 is a single transmembrane protein containing a luminal EF-hand that acts as a Ca^2+^-sensing domain and a cytosolic COOH strand, which is localized in the membrane of the endoplasmic reticulum, the DTS or the acidic Ca^2+^ stores in platelets, respectively [37]. Consequently, STIM1 function is crucial for platelet activation and thrombus formation. In 2007 Grosse and colleagues demonstrated that mice carrying an activating EF-hand mutation in STIM1 have a macrothrombocytopenia and display a severe bleeding phenotype [10]. Platelets from these mice show a decreased life span due to a preactivation state represented by increased basal Ca^2+^ concentration and activated α_IIb_β_3_ integrin levels [10]. This pre-active state and decreased lifespan translated into impaired ITAM signaling and collagen-induced thrombus formation in platelets with a hyperactive STIM1 molecule, when compared to wild-type platelets [10]. In accordance with these observations, a genetic deficiency of STIM1 in platelets resulted in a markedly impaired platelet Ca^2+^ influx upon activation with GPVI- or GPCR agonists, which finally culminated in a decreased in vitro and in vivo thrombus formation [38]. Thereby, the observed STIM1-associated phenotype was accompanied by a mild bleeding phenotype, although the thrombin-dependent aggregation was unaffected in platelets from STIM1-deficient mice when compared to wild-type cells [38]. Moreover, STIM1-associated SOCE in platelets seems to be essential for the initial pro-coagulant activity and thus the first wave of thrombin generation but not the subsequent pro-adhesive function of platelets [39]. Altogether, these results demonstrate that the intracellular Ca^2+^ mobilization from the DTS in platelets is sufficient to induce the initial α_IIb_β_3_ integrin activation, whereas sustained integrin activation needed for stable thrombus formation under flow requires SOCE-mediated Ca^2+^ rises. Of note, the importance of STIM1 for platelet function was substantiated by human patient samples carrying mutations in the STIM1 protein. In 2013 a female patient with a homozygous R429C mutation in STIM1 was described in a case report [40]. The R429C mutation in STIM1 results in impaired cytoplasmic STIM1 oligomerization and abolishes STIM1-ORAI1 interactions [41], thus possibly leading to the observed platelet secretion defect of the α- and δ-granula in the above described case report [40], which pinpoints the importance of STIM1 for the pathogenesis of thrombo-occlusive disorders in humans. As a matter of fact, in 2018 platelets from a patient with the R429C mutation showed a regular activation-dependent Ca^2+^ influx after GPCR or GPVI stimulation, whereas the SOCE after thapsigargin induction was completely abolished [23].

The 4-transmembrane-spanning pore-forming calcium release-activated channel (CRAC) moiety Orai1 is expressed in the plasma membrane of platelets [9,35,36], which forms oligomeric CRAC complexes upon activation. Using a quantitative RT-PCR approach, Tolhurst et al. showed in 2008 that Orai1 is the most prominent Ca^2+^ permeable ion channel in platelets and megakaryocytes when compared with the expression levels of TRPC1, TRPC6 and TRPM2 [42]. Although Orai1-3 isoforms are expressed in platelets and megakaryocytes, Orai1 was confirmed as a major CRAC in platelets, since platelets from mice with an inactive mutation in Orai1 (R93W) showed an impaired α_IIb_β_3_ integrin activation, P-Selectin exposure and a vastly diminished pro-coagulant activity as demonstrated by PS surface exposure [35]. Remarkably, while genetic inactivation of Orai1 is not sufficient to affect platelet aggregation and thrombus formation [35], a complete genetic deficiency of Orai1 results in a highly reduced GPVI-dependent platelet aggregation and thrombus formation, which could not be compensated by the Orai2 or Orai3 isoforms [9]. These results were substantiated in platelets from a patient carrying a homozygous Orai1 mutation. While this patient showed regular Ca^2+^ store depletion upon GPCR or GPVI stimulation, the Ca^2+^ influx was substantially reduced only in a GPVI-dependent fashion and the thapsigargin-induced SOCE was completely abolished [23], which is similar to the observations in Orai1-deficient platelets [9]. Additionally, the pro-coagulant activity in these platelets was markedly reduced, whereas the α_IIb_β_3_ integrin activation and P-Selectin exposure were unaffected [23]. Although, the roles of Orai2 and Orai3 in platelet function were never studied in detail, there is evidence that stimulus-specific Ca^2+^ store depletion in platelets results in exclusive STIM-CRAC complexes involving STIM1 and STIM2 as well as Orai1 and Orai2, while Orai3 associates with TRPCs in a non-capacitive manner [36]. Indeed, so far, an Orai3-dependent CRAC has only been described in estrogen receptor-positive breast cancer cells [43,44]. Of note, although mice lacking Orai2 are protected from ischemic neuronal death in a murine model of ischemic stroke, this effect was independent of the hematopoietic system and the tail bleeding time was unaffected in these mice [45].

Besides the basic components, STIM1 and Orai1, platelets utilize a wide variety of molecular signaling pathways to fine-tune the activation-dependent SOCE with subsequent platelet activation and thrombus formation.

## 4. Molecular Regulation of SOCE in Platelets

**Figure 2:**
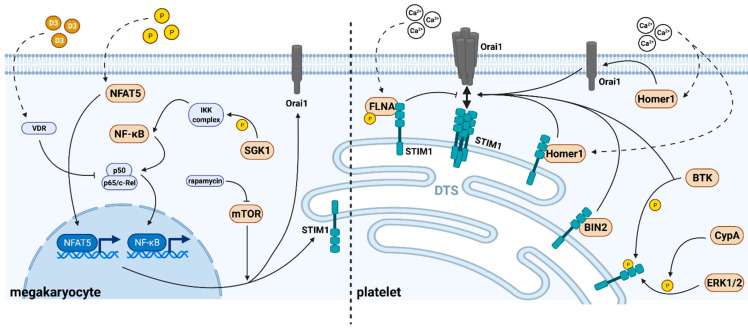
Molecular regulation of the SOCE in megakaryocytes and platelets. Left: In megakaryocytes, the expression levels of STIM1 and Orai1 are regulated by transcriptional processes. Extracellular phosphate (P) activates NFAT5-dependent transcription, while SGK1 and 1,25(OH)_2_D3 (D3) affect the NF-κB-dependent transcription via the IκB kinase (IKK) complex or the vitamin D receptor (VDR), respectively. Since mTOR affects translation processes, both transcriptional and translational regulation in megakaryocytes determine the final protein levels of STIM1 and Orai1 in platelets. Right: In platelets, the SOCE is mainly affected by direct protein-protein interactions or posttranslational modifications of STIM1 and Orai1 which subsequently regulate the interaction of the subunits and finally the formation of the CRAC.

Although STIM1 and Orai1 have been known as the major SOCE in platelets for just a bit more than a decade, there is a growing body of the literature in the meanwhile unraveling molecular regulation mechanisms of SOCE in platelets. In general, the SOCE of platelets can be modulated by transcriptional regulation of its components in megakaryocytes, by direct protein-protein interactions or by post-translational modifications such as protein phosphorylation of STIM1 and Orai1 (Figure 2 and Table 1).

### 4.1. Transcriptional Regulation of STIM1 and Orai1 in Megakaryocytes

NF-κB: The nuclear factor kappa-light-chain-enhancer of activated B cells (NF-κB) is expressed in almost all cell types and plays pivotal roles in stress-induced protein transcription and immune responses. It is known to be involved in megakaryopoiesis and megakaryocytic gene expression and thus plays an important role in platelet function [46,47]. Of note, genes regulated by NF-κB in mast cells include both, STIM1 and Orai1 and HEK293 cells transfected with NF-κB subunits p65/p50 or p65/p52 displayed increased levels of STIM1 and Orai1 [48]. As demonstrated by a work from Borst and colleagues, a NF-κB-dependent upregulation of Orai1 in megakaryocytes and subsequently platelets was confirmed [8]. Finally, this work clearly implicated the role of NF-κB-induced protein transcription in megakaryocytes with platelet SOCE and function upon platelet activation [8].

1,25(OH)_2_ vitamin D3: 1,25(OH)_2_ vitamin D3 (1,25(OH)_2_D3) is the biologically active metabolite of Vitamin D and is a known regulator of NF-κB function by affecting the NF-κB subunits p65/cRel and p50 [49,50]. 1,25(OH)_2_D3 formation is inhibited by fibroblast growth factor 23 (FGF23) and its corresponding receptor together with the type-I membrane protein Klotho which acts as co-receptor for the fibroblast growth factor receptor [51]. Accordingly, mice with a Klotho deficiency inherit pathophysiological elevated 1,25(OH)_2_D3 plasma levels, which can be normalized by dietary vitamin D restriction [52]. Remarkably, megakaryocytes and platelets from Klotho-deficient mice displayed significantly decreased STIM1 and Orai1 transcript as well as protein levels which resulted in diminished activation-dependent SOCE in platelets with subsequent impaired platelet activation [53]. Similarly, treatment of megakaryocytes with 1,25(OH)_2_D3 reduced CRAC currents in these cells significantly and a low 1,25(OH)_2_D3 diet reversed these effects in megakaryocytes and platelets from Klotho-deficient mice [53]. Thereby, the effect of 1,25(OH)_2_D3 on platelet function and thrombus formation could clearly be linked to NF-κB-dependent transcriptional mechanisms, since Klotho-deficiency resulted in an impaired p65/cRel nuclear translocation and a decreased p50 expression in megakaryocytes [53].

NFAT5: The nuclear factor of activated T-cells 5 (NFAT5) transcription factor is mainly described in inducible gene transcription during immune responses but is also expressed in megakaryocytes [54]. NFAT5 is upregulated, for instance, by high phosphate levels in plasma and tissue as prevalent during chronic kidney disease [55], a disease, which is also directly linked to cardiovascular events [56]. Treatment of a human megakaryocytic cell line (Meg01) with the phosphate-donor ß-glycerophosphate mimicking enhanced extracellular phosphate resulted in increased expression levels of STIM and Orai isoforms and consequently in an elevated Ca^2+^ entry after thapsigargin stimulation in these cells [54]. Interestingly, in accordance with these observations, platelets from patients with chronic kidney disease displayed a significantly increased NFAT5 and Orai1 expression as well as protein levels, whereas STIM1 expression only showed a tendency of elevation when compared to platelets from healthy volunteers [54].

mTOR: The mammalian Target of Rapamycin (mTOR) is a serine/threonine protein kinase that regulates pivotal cellular functions including cell proliferation, cell motility, cell survival, protein synthesis and transcription. mTOR is known to regulate megakaryocyte proliferation and differentiation as well as platelet function [57,58]. At least in pulmonary arterial smooth muscle cells and mouse embryonic fibroblasts, mTOR was described as a positive regulator of STIM1 and Orai1 expression [59,60]. However, just lately, mTOR was also linked to STIM1 and Orai1 protein levels in megakaryocytes and platelets. Analyzing platelets from Collagen VI-deficient mice that harbor a hyperreactive mTOR complex revealed increased STIM1 and Orai1 protein levels and an enhanced activation-dependent platelet function [61]. Interestingly, mTOR inhibition by rapamycin treatment in these cells normalized STIM1 and Orai1 expression as well as restored normal platelet SOCE and activation [61]. This study points to a role of mTOR in the regulation of STIM1 and Orai1 expression levels in megakaryocytes and subsequently in platelet SOCE and thrombus formation, although further work is needed to unravel the detailed underlying mechanism.

### 4.2. Regulation of STIM1 and Orai1 in Platelets

#### 4.2.1. Regulation of STIM1

BIN2: The bridging integrator (BIN) family of adaptor proteins consists of three members, regulating a wide variety of cellular functions. The BIN2 isoform is mainly expressed in hematopoietic cells [62] and was described as a mediator of monocyte/macrophage and mast cell function in the first place [63]. By employing a matrix affinity column approach with subsequent proteomic analysis and immunoprecipitation assays, BIN2 was also recently identified to be expressed in platelets and to act as STIM1 interaction partner [64]. In the same study, a pivotal effect of BIN2 on STIM1/Orai1-mediated SOCE in platelets was nicely demonstrated, as BIN2-deficient platelets had a similar Ca^2+^ store content and thapsigargin-induced Ca^2+^ store release, but show an attenuated extracellular Ca^2+^ influx upon thapsigargin treatment or receptor-associated platelet activation with most of the known agonists when compared to wild-type platelets [64]. As shown in a more detailed set of experiments, Volz et al. also demonstrated an IP_3_R-dependent effect of BIN2 on Ca^2+^ fluxes, since BIN2 also interacts with the IP_3_R. In line with these findings, platelets from BIN2-deficient mice showed an impaired (hemi)ITAM-dependent platelet α_IIb_β_3_ integrin activation, P-Selectin exposure and aggregation, which finally translated into disrupted thrombus formation [64]. Despite the remarkable role of BIN2 in the STIM1/Orai1-driven SOCE in platelets, further studies are needed to unravel the exact underlying mechanisms or to define other involved proteins.

Filamin A (FLNA): Filamin A is an actin-cross-linking protein located in the cytoskeleton underneath the plasma membrane where it is known to regulate receptor clustering and receptor/actin cytoskeleton cross talk [65]. Remarkably, patients with a mutation in the X-linked FLNA gene display an abnormal megakaryocyte differentiation leading to a macrothrombocytopenia and altered platelet morphology resulting in common hemorrhages and coagulopathies [66]. In line with these observations, platelets from mice lacking FLNA failed to spread and showed a decreased α-granule secretion and α_IIb_β_3_ integrin activation upon induction of (hemi)ITAM signaling [67]. In 2017 Lopez and colleagues demonstrated an interaction of FLNA and STIM1 as a result of Ca^2+^ store depletion in platelets [68]. In this study, as shown by immunoprecipitation experiments, FLNA was defined as a negative regulator of platelet SOCE by the Ca^2+^-dependent interaction with STIM1. Accordingly, knockdown of FLNA by means of siRNA techniques resulted in increased SOCE in platelets and blocking of the STIM1/FLNA interaction by an anti-FLNA antibody enhanced platelet aggregation upon thrombin stimulation [68]. Of note, the interaction of FLNA and STIM1 in platelets was also crucially dependent on the phosphorylation of FLNA at Ser^2152^, which in return regulates the localization of STIM1 within the cytoskeletal fraction and its interaction with Orai1 [68]. FLNA was reported to be phosphorylated by the protein kinase A (PKA) a long time ago [69,70] and PKA-dependent phosphorylation of FLNA was associated with platelet aggregation in the early 1980s [71].

Homer1: Homer proteins represent a family of adaptor proteins that primarily interact with Ca^2+^-binding proteins [72]. The isoform Homer1 is a well-established mediator of IP_3_R-TRPC interaction [73], but was also shown to interact in a Ca^2+^-dependent manner with Orai1 and in particular STIM1 in platelets upon thapsigargin and thrombin stimulation [72]. In co-immunoprecipitation assays, Homer1 interacted with STIM1, which includes the Homer1 consensus site PxxF [74], and to a weaker extend also with Orai1 [72]. Interestingly, these interactions were only observed in the presence of cytosolic Ca^2+^, and the Homer1-Orai1 interaction was mediated by STIM1 [72]. Perturbation of Homer1 function finally results in an impaired thrombin-evoked Ca^2+^ influx in platelets and a reduced platelet aggregation [72]. However, the detailed role of Homer1 in activation-dependent SOCE in platelets with subsequent thrombus formation still has to be determined.

ERK1/2: Extracellular signal-regulated kinases (ERKs) are MAP kinases (MAPK) and used in a wide variety of cells as crucial intracellular signaling molecules. MAPK and in general ERK1/2 are long known to be highly expressed in platelets [75,76] and to mediate a plurality of platelet functions such as adhesion, granule secretion and aggregation [77]. For several years ERK1/2 has been known to affect Ca^2+^ store depletion with a subsequent extracellular Ca^2+^ influx in platelets, although the underlying mechanism was not clearly understood [78]. However, only in 2010 it was demonstrated in a cell culture model using HEK293 cells, that ERK1/2 directly phosphorylates STIM1 and therefore affects its ability to induce SOCE [79]. Shortly afterwards, the effect of ERK1/2 on activation-dependent SOCE was confirmed in platelets by Elvers et al. [80]. Since ERK1/2-dependent phosphorylation of STIM1 was not important for the association of STIM1 and Orai1, an ERK signaling pathway-dependent interaction of STIM1 with the sarcoendoplasmic reticulum ATPase (SERCA2b) was postulated [80], but the identification of the exact mechanism needs further investigations. However, in line with this work, it could also be unraveled that the ubiquitously expressed peptidyl-prolyl cis-trans isomerase activity-containing chaperone cyclophilin A (CypA) supports the ERK1/2-dependent phosphorylation of STIM1 and thus affects platelet SOCE [80]. Phosphorylation of STIM1 was abrogated in platelets from CypA-deficient mice, and the activation-dependent Ca^2+^ fluxes were significantly diminished in platelets lacking CypA when compared to wild-type cells [80]. Consequently, CypA deficiency compromised platelet function and thrombus formation in vitro and in vivo [80].

BTK: Bruton’s tyrosine kinase (BTK) is a tyrosine kinase that was initially described as a crucial player in B cell development but is well-established as part of platelet activation and thrombo-inflammatory processes [81,82]. BTK has been known to affect platelet SOCE since 2005, when Redondo and co-workers showed that platelet stimulation with thapsigargin or thrombin in human platelets resulted in a rapid activation of BTK independently of intracellular Ca^2+^ concentrations [83]. As a consequence, actin filament reorganization as an early hallmark of platelet activation was significantly diminished in the presence of a pharmacological inhibitor of BTK in human platelets [83,84]. Importantly, pharmacological inhibition of BTK turned down the thapsigargin-induced phosphorylation of STIM1, which finally proceeded in a weakened STIM1/Orai1 interaction in the presence of thapsigargin as demonstrated mainly by co-immunoprecipitation assays [85]. In this regard, at least in other cells, tyrosine phosphorylation of STIM1 was described to affect SOCE by mediating STIM1 puncta formation, Orai1 recruitment and STIM1/Orai1 interaction [86,87]. Nevertheless, besides BTK, other tyrosine kinases are surely involved in STIM1 regulation [86], and the exact role of these in activation-dependent platelet SOCE needs further investigations.

#### 4.2.2. Regulation of Orai1

SGK1: The serum and glucocorticoid-regulated kinase 1 (SGK1) is a cytosolic serine/threonine kinase downstream of the phosphoinositide 3-kinase (PI3K), which is, together with its downstream effectors, a critical signaling molecule in thrombopoiesis and platelet activation [12,88,89]. SGK1 is upregulated by the transcription factor NFAT5 [90] and has been reported to mediate the function of several ion channels and carriers in the plasma membrane of cells [91]. SGK1 function is highly affected by gluco- and mineralocorticoids [92] and SGK1 is highly expressed in megakaryocytes and platelets [8]. At least in megakaryocyte SGK1 acts as an important transcriptional regulator of NF-κB function, and thus Orai1 expression, by phosphorylation of the IκB kinase α/β with subsequent degradation of the NF-κB inhibitory protein IκB and nuclear NF-κB translocation [8]. Therefore, megakaryocytes and platelets isolated from SGK1-deficient mice show significantly decreased Orai1 protein levels, thapsigargin-induced SOCE as well as activation-dependent extracellular Ca^2+^ influx [8]. As a consequence, although SGK1-deficiency does not fully abrogate Ca^2+^ signaling and especially Ca^2+^ store release in platelets, platelets from SGK1-deficient mice exhibit decreased pro-coagulant activity, platelet secretion, platelet migration, aggregation and thrombus formation in a GPVI-dependent fashion [8,93].

Remarkably, besides NF-κB/SGK1 and Homer1 there are no other established direct regulators of Orai1 in platelets. Although further investigations are required to identify novel interaction partners of Orai1 in platelet activation and thrombus formation, there are studies raising the possibility of other proteins involved in Orai1-mediated SOCE in platelets.

For instance, based on work in other cell types, lipid rafts are known to affect Orai1 regulation [94], and in platelets lipid rafts are described as important mediators of SOCE components on the platelet surface [95,96]. For a long time, lipid rafts have been known to regulate the assembly and activity of plasma membrane-located ion channels and carriers and that the properties of lipid rafts are crucially dependent on the presence of cholesterol in the plasma membrane [97,98]. Interestingly, there is ample evidence that chemically induced cholesterol depletion enhances SOCE and Orai1 currents at least in HEK293 and rat basophilic leukemia mast cells [98]. Further data substantiated the interplay between Orai1 and cholesterol, since single point mutations in the amino terminus of Orai1 abolished cholesterol binding and enhanced SOCE [98]. Taken together, these findings raise the possibility of a lipid-raft-dependent Orai1 regulation during platelet activation, at least in theory. Nevertheless, a detailed study is required to investigate the implication of lipid rafts in Orai1 regulation in platelets.

Another possible mediator of Orai1 function in platelets could be stanniocalcin 2 (STC2), which was described in 2002 as a protein involved in phosphate and calcium homeostasis in mammals [99]. In fibroblasts and a hippocampal cell line, STC2 deficiency resulted in an elevated Ca^2+^ influx upon store depletion, pointing to a negative regulation of SOCE by STC2 [100]. In accordance with this study, platelets from STC2-deficient mice showed an attenuated STIM1/Orai1 interaction as demonstrated by immunoprecipitation assays, but surprisingly also displayed an enhanced SOCE upon thrombin stimulation [101]. Thus, thrombin as well as ADP stimulation triggered a reinforced platelet aggregation in STC2-deficient platelets and resulted in a significantly decreased tail bleeding time in STC2-deficient mice when compared with wild-type littermates [101]. Surprisingly, platelets lacking STC2 seemed to have an increased Orai3 expression thus pointing to a STC2-mediated non-capacitive Ca^2+^ entry mechanism [101]. Although a STC2-dependent regulation of STIM1/Orai1-induced SOCE in platelets is very likely, further investigations have to be performed to unravel the implication of STC2 in activation-dependent SOCE in platelets and to clarify the still existing discrepancies.

## 5. Pathophysiological Implications of Platelet SOCE in Thrombo-Occlusive Disorders and Hemostasis

The activation of platelets with subsequent platelet aggregation and thrombus formation is a major mechanism underlying thrombo-occlusion and ischemia/reperfusion injury. Thereby, as demonstrated in a plurality of genetic in vivo mouse models, the SOCE-mediated intracellular Ca^2+^ levels in platelets play a crucial role in the formation of pathological thrombus formation, whereas the observed effects are crucially dependent on the techniques of thrombus induction. While mechanically induced vessel damage is mainly dependent on collagen/GPVI-mediated platelet signaling, the commonly used chemical-induced (FeCl_3_) arterial thrombus formation is highly thrombin/GPCR dependent. Nevertheless, STIM1 or Orai1 deficient mice especially seem to be protected from arterial thrombus formation [9,38,39].

STIM1-deficient chimeric mice display a regular tail bleeding time but present a decreased thrombus formation and a prolonged time to vessel occlusion in a mechanically- and chemically-induced in vivo mouse model of arterial thrombosis [38]. In doing so, at least part of this effect was due to decreased pro-coagulant activity of STIM1-deficient platelets, which finally resulted in impaired thrombus stability [38]. In addition, when compared to wild-type littermates, STIM1-deficient chimeric mice showed a substantially reduced infarct size and a better neurological outcome without intracranial hemorrhages in a transient middle cerebral artery occlusion (tMCAO) mouse model of ischemic stroke [38]. Remarkably, these STIM1-dependent effects on in vivo thrombus formation seem to be mediated at least in part by the influence of immune cells, since in vitro thrombus formation of megakaryocyte/platelet-specific STIM1-deficient mice was comparable with their wild-type littermates [39]. After laser injury in cremaster muscle arterioles in megakaryocyte/platelet-specific STIM1-deficient mice, the initial thrombus formation was unchanged, but the thrombus was highly unstable as depicted by a decreased fibrin generation and impaired PS exposure [39], underpinning the importance of STIM1 signaling for pro-coagulant platelet activation and thrombus stability [102]. In contrast to these antithrombotic effects of STIM1, the decreased vessel occlusion observed in mice bearing an EF hand mutation in STIM1 (STIM1^Sax/+^) is most likely triggered by the thrombocytopenia present in these mice [10]. Unlike chimeric or megakaryocyte/platelet-specific STIM1-deficient mice, STIM1^Sax/+^ mice also show prolonged bleeding times [10], which is in accordance with phenotypes observed in patients with a gain of function mutation in the STIM1 [23,103,104,105].

Similar to STIM1-deficient chimeric mice, chimeric mice with an Orai1 deficiency show a regular tail bleeding time and are highly protected from collagen/epinephrine-induced pulmonary embolism [9]. Interestingly, these Orai1-deficient mice are protected only from thrombus formation after collagen/GPVI-dependent mechanical vessel injury and display a similar thrombus formation after FeCl_3_-induced arterial thrombus formation when compared to wild-type mice, which is in close accordance to the results gained from in vitro platelet function assays [9]. Accordingly, Orai1-deficiency results in a minimized infarct volume and an improved clinical outcome after induction of the tMCAO ischemic stroke mouse model without any sign of intracranial hemorrhagic bleeding [9]. These results were nicely supported by work from Nagy et al. in 2018, which investigated blood samples from patients with a gain of function mutation in Orai1 [23]. Performing in vitro flow chamber experiments on collagen, rhodocytin and fibrinogen-coated, it was shown that a gain of function Orai1 mutation results in significantly impaired in vitro thrombus formation, most likely due to an impaired PS exposure and pro-coagulant platelet activity [23].

The importance of platelet SOCE for pathological thrombosis with subsequent vessel occlusion and tissue ischemia is further substantiated by a plurality of studies unraveling the effect of the molecular regulators of STIM1/Orai1-mediated Ca^2+^ signaling in platelets. The NF-κB-mediated transcription of STIM1 and Orai1 is a pivotal mechanism of SOCE signaling in platelets since NF-κB-affecting proteins and compounds such as SGK1 and 1,25(OH)_2_D3 pivotally affecting platelet function and thrombus formation. Notably, the tail bleeding time in SGK1-deficient mice was unaffected [8], but FeCl_3_-triggered thrombus formation in mesenteric arterioles was significantly delayed in these mice when compared to wild-type littermates [106]. In this regard, atherothrombotic complications such as myocardial infarction or ischemic stroke are often associated with type 2 diabetes mellitus [107], which is known to be highly linked with hyperglycemia and advanced glycation end products (AGEs). Both, excessive glucose concentrations as well as AGEs are powerful stimulators of SGK1 expression and function [108]. Consequently, the SGK1 mediated elevation in SOCE-dependent Ca^2+^ entry in platelets could explain the enhanced platelet activation and thrombus formation in diabetic platelets [107]. Besides SGK1, low 1,25(OH)_2_D3 levels are associated with an increased risk of thrombo-occlusive disorders as shown in a meta-analysis of a large consortium of cohort studies in 2014 [109], although a monthly high-dose vitamin D supplementation has no effect on the occurrence of cardiovascular diseases [110]. However, a low vitamin D diet normalized GPVI-dependent arterial in vitro thrombus formation in mice with elevated 1,25(OH)_2_D3 plasma levels, which normally showed a drastically impaired in vitro thrombus formation when compared with mice showing physiological 1,25(OH)_2_D3 levels [53]. Thus, at least in part, the demonstrated inhibitory effect of 1,25(OH)_2_D3 on SOCE-mediated platelet activation could explain the high prevalence of thrombotic cardiovascular disorders in humans with low vitamin D levels.

The adaptor protein BIN2 was recently identified as part of the platelet activation machinery [64]. Megakaryocyte/platelet-specific deletion of BIN2 only affected hemostasis in a TxA_2_-dependent fashion, since tail bleeding time in these mice was only impaired in the presence of aspirin, confirming that the functional defect in (hem)ITAM-signaling can be compensated by TxA_2_ in vivo [64]. More importantly, the time to vessel occlusion in a mechanical injury model of the abdominal aorta was significantly prolonged in BIN2-deficient mice when compared with wild-type animals [64]. In line with this observation, lack of BIN2 resulted in highly reduced infarct volumes and decreased occluded vessels in the ipsilateral hemisphere in a tMCAO mouse model of ischemic stroke [64]. Together, these results clearly pinpoint BIN2 as an important player in arterial GPVI-dependent thrombosis and ischemic stroke with subsequent brain infarction and tissue damage.

Besides SGK1 and BIN2, the molecular SOCE regulators FLNA and CypA are also well-described in pathological thrombus formation. FLNA has been especially appreciated for a long time as a crucial mediator of platelet function and was recently connected to SOCE signaling in platelets [68]. Interestingly, deletion of FLNA in the erythroid/megakaryocytic lineage results in a significantly impaired platelet adhesion under high arterial shear rates [67]. The observed effect was highly dependent on the VWF signaling pathway [67], which is one of the most prominent mediators of FeCl_3_-induced thrombus formation. Since FLNA deficiency in platelets results in an increased tail bleeding time and a severe thrombocytopenia [111], the perturbated platelet adhesion on VWF could be due to a decreased platelet number. However, normalization of platelet counts in blood from patients with a truncated FLNA protein lead to a decreased thrombus growth on collagen too [112]. In 2012, the chaperone CypA was also described as a major player in platelet activation and arterial thrombus formation [80]. Mice lacking the intracellular CypA display a regular tail bleeding time and a normal initial thrombus formation in an in vivo mouse model of FeCl_3_-induced arterial thrombus formation when compared with wild-type littermates [80]. The time to vessel occlusion was highly prolonged in CypA-deficient mice though [80], which points to a CypA-dependent effect on thrombus stability that is similar to the observations after STIM1 or Orai1 deletion.

## 6. Implications for Potential Pharmacological Interventions

To date, despite a high anti-thrombotic efficiency, the vast majority of clinically available antiplatelet and antithrombotic compounds are still associated with a lack of efficacy or serious side effects such as life-threatening bleeding complications. Considering their importance for platelet activation and pathological thrombus formation in vivo, STIM1 and Orai1, as well as their molecular regulators, represent a promising target for the pharmacological treatment of thrombo-occlusive disorders. In particular, molecular targets that do not, or only mildly affect bleeding times, are promising candidates for improved future anti-platelet and antithrombotic treatment regimens. While there is a plurality of SOCE interfering compounds which are well summarized by Shawer et al. [113], only inhibitors of SOCE compounds described in platelets and thrombus formation are briefly described here.

One of the most common inhibitors of SOCE is 2-Aminoethoxydiphenyl borate (2-APB) compound. Initially described as antagonist of IP_3_ receptors, 2-APB is in the meanwhile a well-established inhibitor of SOCE channels in the plasma membrane [114]. Just recently, 2-APB was identified as a potent inhibitor of PS-exposure and pro-coagulant activity of platelets after treatment with the Ca^2+^ ionophore, A23187, or the pore-forming toxin, streptolysin-O [115]. This observation is in accordance with the importance of SOCE-mediated PS-exposure for thrombus formation and stability in STIM1- and Orai1-deficient mice [9,38]. Although 2-APB protects from severe ischemic stroke in a mouse model of tMCAO [116] and was described to interfere with the STIM1/Orai1 coupling [117], the underlying mechanism is not well-defined and most likely includes additional processes that are independent of SOCE signaling in platelets. 2-APB and the imidazole antimycotic drug SKF-96365 are known to inhibit platelet migration [118]. Although SKF-96365 is described as an inhibitor of SOCE-related Ca^2+^ fluxes in a wide variety of cells, its inhibitory effect was identified in human platelets a long time ago [119]. Moreover, in vivo administration of SKF-96365 leads to a significantly reduced atherosclerotic plaque progression in a mouse model using apolipoprotein E knockout mice [120]. However, comparable with 2-APB, the effect of SKF-96365 on platelet function and atherothrombosis cannot be clearly linked with the SOCE machinery in platelets, since a lot of other ion channels are reported to be affect by SKF-96365 [113]. Amongst others, work by Derler and colleagues in 2013 identified GSK-7975 A as a pore-forming blocking compound of Orai-mediated SOCE [121]. As a matter of fact, GSK-7975 A was shown to inhibit pro-coagulant activity of platelets and affecting thrombus formation as potently as 2-APB [116]. Another GSK small molecule designated GSK-650394 is an established specific SGK1 inhibitor [122]. Accordingly, treatment of the megakaryocytic cell line Meg-01 with 1 µM GSK-650394 abolished the SGK1-induced upregulation of Orai1 mRNA, protein levels in the plasma membrane and subsequently thapsigargin-induced SOCE [8]. This observation, together with the fact that SGK1-deficient mice only display a mildly prolonged tail bleeding time [8], thus nicely substantiates a possible use of clinically approved SGK1 inhibitors for thrombo-occlusive disorders.

Notably, since SOCE is an important mechanism for several cells and impacts different cellular processes, platelet-specific treatment with SOCE inhibitors is still very limited due to multiple severe side effects. For instance, global genetic deletion of STIM1 and Orai1 in mice results in an elevated perinatal lethality and the surviving mice show a decreased life span [9,38]. Moreover, in humans, the absence of functional STIM1 or Orai1 proteins converge in life-limiting severe immune system defects [23]. Thus, although SOCE inhibition is a powerful anti-thrombotic mechanism in vitro and in vivo, more thorough investigations are unavoidable to unravel the detailed effect of available SOCE inhibitors on platelet function and pathological thrombus formation.

## 7. Concluding Remarks

Studies and results from the last decade identified SOCE-dependent mechanisms as pivotal factors of platelet activation, aggregation and thrombus formation. Upon activation-dependent Ca^2+^ store release, the extracellular Ca^2+^ influx in platelets is mainly carried out by STIM1 and Orai1, which are the major SOCE components in platelets [9,38]. In recent years, there has also been an increasing body of the literature unraveling interaction molecules and regulators of platelet STIM1 and Orai1.

Adaptor and bridging proteins [64,67,68,72,80] as well as protein kinases [8,78,80,83] are well-established regulators of SOCE in platelets. Atherothrombotic complications of human disorders associated with these regulatory factors such as type 2 diabetes mellitus or filaminopathies, could possibly be explained by increased platelet SOCE and subsequent platelet activation. Indeed, besides STIM1 and Orai1, this could make the SOCE-linked regulatory molecules a suitable target for future antiplatelet and anti-thrombotic therapies

Since STIM1/Orai1-mediated SOCE in platelets is sufficient for thrombus formation without affecting hemostasis, it is a promising target for the treatment of thrombo-occlusive disorders including arterial thrombosis, myocardial infarction and ischemic stroke. Unfortunately, the long-term inhibition of SOCE in the treatment of these disorders is not expedient since perturbation of STIM1/Orai1 also leads to severe immune defects. However, acute pharmacological impairment of SOCE in platelets during pathological thrombosis is potentially conceivable. In any case, further detailed studies are necessary to completely understand the complex signaling mechanisms of SOCE-mediated platelet activation to develop novel specific inhibitors of thrombo-occlusive disorders in humans.

## Figures and Tables

**Table 1:** Described direct regulators of STIM1/Orai1-mediated SOCE in platelets.

STIM1			
Regulator	Function	Effect	Ref.
NFAT5	transcriptional regulation of protein expression	increased STIM1 protein levels in platelets	[54]
NF-κB	transcriptional regulation of protein expression	increased STIM1 protein levels in platelets	[8]
1,25(OH)_2_ vitamin D3	NF-κB-dependent transcriptional regulation of protein expression	decreased STIM1 protein levels	[53]
mTOR	translational regulation	increased STIM1 protein levels	[61]
BIN2	STIM1 interaction	improved STIM1/Orai1 coupling	[64]
FLNA	STIM1 interaction	STIM1 localization and negative regulation of STIM1/Orai1 coupling	[68]
Homer1	STIM1 interaction	improved STIM1/Orai1 coupling	[72]
ERK1/2	STIM1 phosphorylation	improved SERCA2b/STIM1 coupling	[78,80]
CypA	chaperone for ERK1/2-mediated STIM1 phosphorylation	improved SERCA2b/STIM1 coupling	[80]
BTK	STIM1 phosphorylation	improved STIM1/Orai1 coupling	[83,84]
**Orai1**			
**Regulator**	**Function**	**Effect**	**Ref.**
NFAT5	transcriptional regulation of protein expression	increased Orai1 protein levels in platelets	[54]
NF-κB	transcriptional regulation of protein expression	increased Orai1 protein levels in platelets	[8]
1,25(OH)_2_ vitamin D3	NF-κB-dependent transcriptional regulation of protein expression	decreased STIM1 protein levels	[53]
mTOR	translational regulation	increased STIM1 protein levels	[61]
SGK1	regulation of NF-κB-dependent protein expression in megakaryocytes	increased Orai1 protein levels in platelets	[8]
Homer1	Orai1 interaction	improved STIM1/Orai1 coupling	[72]

## Data Availability

All presented data are outlined within the manuscript or the cited references.
